# Supplementary material for: What does it take to learn the rules of RNA base pairing? A lot less than you may think
Source: bioRxiv. 2025 Aug 2:2025.07.31.668042. Preprint. [Version 1] doi: 10.1101/2025.07.31.668042 (PMC12324431; doi:10.1101/2025.07.31.668042)
Supplement: Supplement 1 [file media-1.gz › supplemental_material/src/rscape_v2.6.0.tar.gz!/rscape_v2.6.0/lib/tornado/documentation/TORNADO_userguide.pdf]

# TORNADO User's Guide

Version v0.9.1; July 2025

Elena Rivas
elenarivas@fas.harvard.edu
Department of Molecular and Celullar Biology
Harvard University
16 Divinity Avenue
Cambridge MA 02138 USA
http://rivaslab.org/

Copyright (C) 2016 Howard Hughes Medical Institute.

Permission is granted to make and distribute verbatim copies of this manual provided the copyright notice and this permission notice are retained on all copies.

TORNADO is licensed and freely distributed under the GNU General Public License version 3 (GPLv3). For a copy of the License, see http://www.gnu.org/licenses/.

# Contents

**1 Introduction** **3**

**2 Installation** **3**

**3 How to write an RNA grammar in TORNADO language** **3**
A simple example . . . . . . 4
General principles of the TORNADO language . . . . . . 5
Specific details to write a grammar in TORNADO language . . . . . . 7
Detailed description of a particular grammar: ViennaRNAG . . . . . . 12

**4 Inference programs implemented in TORNADO** **18**
Obtaining properties of and RNA grammar and debugging tool: `grm-parse` . . . . . . 18
Training: `grm-train` . . . . . . 19
Testing: `grm-fold` . . . . . . 19
Comparing a trusted with a predicted structure: `esl-compstruct` . . . . . . 21
Calculate the score (or log probability) of a sequence/structure pair: `grm-score` . . . 22
Sampling suboptimal structures from a probabilistic grammar: `grm-psample` . . . . 22
Emitting sequence/structure pairs from a probabilistic grammar: `grm-emit` . . . . . 22

# 1 Introduction

The code documented in these pages is the companion to manuscript *"A range of complex probabilistic models for RNA secondary structure prediction that include the nearest neighbor model and more"* by E. Rivas, R. Lang, and S.R. Eddy, August 2011.

TORNADO is a general purpose parser to produce grammars of single-sequence RNA secondary structure. TORNADO is written in C, lex and yacc. Installation instructions are given in Section 2. TORNADO allows to implement a wide range of RNA grammars using a specific language that we describe in Section 3. TORNADO includes most of the inference algorithms usual for single-sequence RNA secondary structure prediction which are described in Section 4.

# 2 Installation

Basic instructions to create the TORNADO executables:

Download **`tornado.tar.gz`** from `http://rivaslab.org/`; unpack it, configure, and make:

```
> tar xf tornado.tar.gz
> cd TORNADO
> ./configure
> make
> make install
```

The newly compiled binaries are in the **`TORNADO/bin`** directory. You can run them from there.
Some configuration options are:

| | | |
|---|---|---|
| **./configure** | **- -enable-debugging** | # debugging implementation. |
| **./configure** | **- -enable-mpi** | # to invoke an MPI implementation. |

Both configuration flags can be used together as well. The same configuration flags should be used both for easel and TORNADO.

# 3 How to write an RNA grammar in TORNADO language

The TORNADO parser includes a lexical interpreter (file grm_parsegrammar.lex) that reads the input file, and a compiler (file grm_parsegrammar.y) that implements a "meta" context-free grammar (the language parser for RNA grammars) and translates the input file for a specific RNA grammar into a generic C structure that can be used by any of the TORNADO inference programs.

## A simple example

SCFGs consist of nonterminals, terminals (the actual residue emissions), and production rules that recursively determine which strings of terminals the grammar permits. A simple example of an RNA grammar in TORNADO language is

```
# g6s [Pfold grammar with stacking]
    S --> L(i,k) S(k+1,j) | L            # Start nonterminal has two rules
    L --> a:i&j        F(i+1,j-1) | a:i # helix starts | one single emission
    F -->a:i&j:i-1,j+1 F(i+1,j-1) | L S # helix continues | helix ends
```

In TORNADO, non-terminals are specified with capital letters, and terminals with lower-case single letters (one letter per emission even if the emission consist of more than one residue). The "g6s" grammar has three nonterminals (`S`, `L`, `F`). Each nonterminal has two rules, for a total of six rules.

Rules for the same nonterminal can be put together with a | (the or symbol) as depicted above, or in separate lines as desired. For instance, an equivalent (albeit less clear) description of the "g6s" grammar is:

```
# g6s [Pfold grammar with stacking]
    S -> L S
    F -> a:i&j:i-1,j+1  F(i+1,j-1)
    L -> a:i
    F -> L S
    L -> a:i&j  F(i+1,j-1)
    S -> L
```

Different rules for the same nonterminal can be given in any order. The only constrain is that the left-had side nonterminal of the first rule will be interpreted as the start nonterminal, (`S` for this grammar).

There are three emitting rules in this grammar, each emitting a different residue type:

- One single residue emission; `a:i`.
- One plain basepair emission: `a:i&j`.
- One stacked pair emission dependent on the two adjacent outside bases: `a:i&j:i-1,j+1`.

  Emitted residues are separated from context residues with a colon, and a basepair is characterized by a "&", to distinguish it from two unpaired bases (for instance a mismatch emission `a:i,j:i-1,j+1`). There can be an arbitrarily large number of emissions and contexts.

In a non-stochastic context-free grammar, each rule gets associated an arbitrary score which might depend on the terminals in the rule. For an SCFG, each rule has associated a "transition" probability so that the sum of the transition probabilities for a given nonterminal is one. For each rule, each terminal corresponds to an "emission" (of one or several residues), and has associated a probability distribution. In this example, the existence of transition and emission distributions is specified implicitly by the rules.

## General principles of the TORNADO language

Description of features allowed by TORNADO:

***4 possible iterators:*** In addition to the left-most 5′ (`i`) and right-most 3′ (`j`) iterators, TORNADO allows up to two intermediate iterators represented by "`k`" or "`l`", such that `i`≤ `k`≤ `l`≤ `j`. The `i,j` (`k,l`) notation establishes a connection with the actual dynamic programming routines that TORNADO will implement for the grammar. These iterators are not necessary for the formal grammar itself, but they simplify the parser without adding much additional complexity. Some simple rules admit simple forms without explicit iterators (like `S -> L` or `S -> LS` in the "g6s" example above), but the form with explicit iterators allows us to describe an arbitrarily large number of complex rules. For instance, a one nt left bulge ($a$) emitted with the closing basepair ($b$, $\hat{b}$) and depending on the previously emitted basepair ($c$, $\hat{c}$) that has the formal grammar notation $\texttt{P}^{c,\hat{c}}$ `->` $a\,b$ `F` $\hat{b}$, in TORNADO adopts the form [$\texttt{P}^{c,\hat{c}}$ `-> a:i,i+1&j:i-1,j+1 F(i+2,j-1)`].

***Production rules:*** can include an arbitrary number of residue emissions, loop emissions, and nonterminals provided that the rule requires no more than four iterators. Examples of possible maximal combinations allowed in TORNADO's rules are: three nonterminals and an arbitrary number of emissions; two nonterminals, one monosegment loop, and an arbitrary number of emissions; one nonterminal, one disegment loop, and an arbitrary number of emissions.

***Arbitrary residue emissions:*** Emissions can include an arbitrary number of residues, and can depend on an arbitrary number of previously emitted residues (contexts). This generalizes the emissions used in the nearest-neighbor model. Typical examples of nearest-neighbor emissions are:

**`Stacked basepairs`** [$\texttt{P}^{c,\hat{c}}$ `->` $a$ `F` $\hat{a}$]: in which a basepair $(a,\hat{a})$ depends on a contiguous basepair $(c,\hat{c})$ (for arbitrary nonterminals `F` and $\texttt{P}^{c,\hat{c}}$).

In TORNADO language: `a:i&j:i-1,j+1 F(i+1,j-1)`.

**`Hairpin mismatches`** [$\texttt{P}^{c,\hat{c}}$ `->` $a$ `[m...m]` $b$]: in which the final two bases of a hairpin loop $(a,b)$ depend on the closing basepair $(c,\hat{c})$.

In TORNADO language: `a:i,j:i-1,j+1 m...m(i+1,j-1)`.

**`Tetraloops depending on closing basepair`** [$\texttt{P}^{c,\hat{c}}$ `->` $a_1\,a_2\,a_3\,a_4$]: Hairpin loops with exactly four bases depending on the closing basepair $(c,\hat{c})$.

In TORNADO language: `a:i,i+1,i+2,i+3:i-1,j+1`.

**`Internal loop mismatches`** [$\texttt{P}^{c,\hat{c}}$ `->` $a$`[d...]`$b$ `F` $\hat{b}$`[...d]`$e$]: where for a internal loop limited by the two basepairs $(c,\hat{c})$ and $(b,\hat{b})$, the closing bases ($a$, $e$) depend on the adjacent basepair $(c,\hat{c})$, and the basepair $(b,\hat{b})$ depends on the adjacent bases in the internal loop.

In TORNADO language: `a:i,j:i-1,j+1 d...(i+1,k)...d(l,j-1) F(k+2,l-2) b:k+1&l-1:k,l`.

**`Left and right dangles`** [$\texttt{P}^{c,\hat{c}}$ `->` $a$ `F | F` $a$]: in which a single left (or right) base depends on the adjacent basepair.

In TORNADO language: `a:i:i-1,j+1 F(i+1,j)` or `b:j:i-1,j+1 F(i,j-1)`.

**`Basepairs depending on left and right dangles`** [$\texttt{P}^{c}$ `->` $a$ `F` $\hat{a}$] [$\texttt{P}^{c,d}$ `->` $a$ `F` $\hat{a}$]: in which a basepair $(a,\hat{a})$ depends on the contiguous unpaired bases ($c$), ($d$), or both.

In TORNADO language: `a:i&j:i-1 F(i+1,j-1)` or `a:i&j:j+1 F(i+1,j-1)` or `a:i&j:i-1,j+1 F(i+1,j-1)`.

Other first order emissions tested with TORNADO, and not included in the standard nearest-neighbor model are:

**dangles in bulges** [ $\mathtt{P}^{c,\hat{c}}$ `->` $a$`[m...m]`$b$ `F` $\hat{b}$]: in which the end base ($a$) of a bulge depends on the adjacent basepair ($c, \hat{c}$), and the closing basepair ($b, \hat{b}$) depends on the adjacent bulge base.

In TORNADO language: `a:i:i-1,j+1 m...m(i+1,k) b:k+1&j:k F(k+2,j-1)`.

**mismatches (or dangles) in multiloops** where multiloop bases contiguous to basepairs depend on the closing basepairs. Details of multiloop dangles are given in Methods.

**coaxial stacking** [ `P ->` $a$ `F` $\hat{a}$ $b$ `F` $\hat{b}$]: where two contiguous stems with closing basepairs ($a, \hat{a}$) and ($b, \hat{b}$) respectively have their final basepair emissions depending on each other.

In TORNADO language: `a:i&k b:j&k+1:i,k F(i+1,k-1) F(k+2,j-1)` or `a:i&k,j&k+1 F(i+1,k-1) F(k+2,j-1)`.

TORNADO can also be used to build second (or higher) order Markov dependencies, rather than just first order. Examples are

**dangles (or more than one single base) depending on several bases** [ $\mathtt{P}^{c,d,e}$ `->` $a$ `F | ` $a$ $b$ `F`]:

In TORNADO language: `a:i:i-1,i-2,i-3 F(i+1,j)` and `a:i,i+1:i-1,i-2,i-3 F(i+2,j)`.

**higher order stacked pairs** [ $\mathtt{P}^{b,\hat{b},c,\hat{c}}$ `->` $a$ `F`$\hat{a}$]:

In TORNADO language: `a:i&j:i-1,i-2,j+1,j+2 F(i+1,j-1)`.

**three single bases depending on two basepairs** [ $\mathtt{P}^{e,\hat{e},f,\hat{f}}$ `->` $a$ $b$ $c$ `F`]:

In TORNADO language: `a:i,i+1,i+2:i-1,i-2,j+1,j+2 F(i+3,j)`.

***Length distributions for loop emission:*** Mono-segment loops (for instance for hairpins, bulges or multiloops), and di-segment loops (for internal loops) can be specified. Disegment loops might include two independent length distributions or a joint one parameterized by the total length of the loop.

***Length distribution tails for loop emissions:*** A length distribution can include a table of specific independent values for lengths up to a value (`p-FIT_LENGTH` in Figure **??**), and a tail (dependent on a small number of parameters) for lengths larger than `p-FIT_LENGTH`. Length distribution tails can be specified in TORNADO in the form of affine (for scores) or geometric (for probabilities) extrapolations.

***Length distributions for stems:*** Base pairs can be emitted as stems of arbitrary lengths governed by a length distribution. Stem length distribution can be combined with stacking emission of the actual basepairs. This feature is a natural addition to the standard nearest-neighbor model.

***Tying of parameters:*** Transitions can be tied internally (so that two rules for the same nonterminal share the same value) or externally (so that two different nonterminals can have the exact same transitions). Emission distributions can also be tied so that for instance a single residue emission (`a:i`) could be a marginalization of a mismatch emission (`a:i,j`), or a mismatch (`a:i,j:i-1,j+1`) could be the product of two independent dangles (`a:i:i-1,j+1`) and (`b:j:i-1,j+1`). A larger list of tying operations for residue emissions has been implemented (see TORNADO's documentation).

***Specific distributions:*** For the purpose of tying parameters, transition, emission and length distributions can be pre-specified as part of the grammar description previous to providing the actual grammar rules.

***Specific values:*** can be assigned to the different distributions as part of the description of the grammar. These values could be free-energy changes obtained from thermodynamic data or arbitrary scores provided by other means. This tasks is helped by the possibility of defining constants that can be interpreted numerically anywhere in the grammar description (and can be defined by mathematical operations), much like the macro definition directive (#define) works in C programming.

***Arbitrary 4x4 canonical basepairs and non-canonical basepairs:*** TORNADO allows distinguishing 18 types of basepairs, depending on the edge (Watson-Crick, Sugar, or Hoogsteen) and the conformation (cis or trans) of the two bases (Leontis and Westhof, 2001). In this work, we only used the canonical basepairing type (Watson-Crick/Watson-Crick in cis) which could involve any of the 4x4 possible residue combinations (or be restricted by design to only G-C, A-U and G-U basepairs).

***Comments:*** can be specified at any time using "#" or "//".

## Specific details to write a grammar in TORNADO language

The actual grammar rules are necessary to describe the grammar. In addition, one can optionally specify before the actual rules, one or more of the following (in the provided order): Arbitrary parameters, transition distributions, emission distributions, and length distributions.

**Arbitrary parameters** *Arbitrary parameters* that might be useful later on in the definition of the grammar. The general description of a parameter definition is:

```
def:  <param_name> :  <param_value>
```

Parameter names have to start with "p-". Parameter values can have dependencies on previously defined parameters. A large number of expressions can be used such as addition, subtraction, multiplication, division, max, min, log, exp, sqrt (square root), sine, cosine, amongst others.

An example:

```
def :  p-GASCONST : 1.98717                         # in [cal/K]
def :  p-K0 :  273.15                               # 0 Celsisus in Kelvin
def :  p-Tmeasure :  37 + p-K0                      # temperature (in Kelvin)
def :  p-kT : p-Tmeasure * p-GASCONST               # k * Tmeasure
def :  p-TT : (p-temperature + p-K0)/(p-Tmeasure)  # if TT ≠ 1 (ie p-temperature ≠ 37),
                                                    # one uses enthalpies,
                                                    # ΔG(T')=T'/T*ΔG(T) + (1-T'/T)*H
                                                    # which comes from ΔG(T) = H - T S
```

```
def :  p-FACTOR : 10.0                        # arbitrary scaling factor
def :  p-SCALE : -p-FACTOR/p-kT               # use this to scale ALL energy parameters
```

**The transition distributions** *Transition distributions* can be pre-specified for tying purposes; otherwise they get defined internally for each nonterminal. Types of tying allowed for transitions are: equating different elements of a given distribution, assigning the same distribution to different (but with identical number of rules) nonterminals, or specifying a particular parameterization of those distributions. Transition distribution names have to start with "t-".

General description of a transition distribution definition is:

```
tdist:  <n> :  <t-name>
```

where <n> is the number of emissions.
An example of a transition distribution with 24 parameters where transitions are set to zero by default, and some have particular values that depend on previously defined parameters is:

```
 # t-P
tdist :  24 :  t-P
td = p-ZERO
0 = p-TT * p-hairpin37_length_3
1 = p-TT * p-hairpin37_length_4
3 = p-TT * p-bulge37_length_1
4 = p-TT * p-bulge37_length_1
5 = p-TT * p-bulge37_length_1
6 = p-TT * p-bulge37_length_1
0 = p-TT * (p-ML_closing37 + p-ML_intern37 + 2*p-ML_BASE37)
21 = p-TT * (p-ML_closing37 + p-ML_intern37 + p-ML_BASE37 + p-coaxial5)
22 = p-TT * (p-ML_closing37 + p-ML_intern37 + p-ML_BASE37 + p-coaxial3)
23 = p-TT * (p-ML_closing37 + p-ML_intern37 + p-coaxial5 + p-coaxial3)
```

If values are specified the default value "`td = `" has to be specified first.

**The emission distributions** *Emission distributions* are specified by providing the number of emissions, contexts, basepairs, and the nature of the basepairs, and the emission name separated by a semicolons. The number of emissions and contexts is in principle unconstrained. Emission names are of the form "e⟨n⟩" where ⟨n⟩ is a natural number. Emissions with different properties (*i.e.* different number of base pairs or emissions or contexts) can use the same name.

General description of an emission distribution definition is:

```
 edist : <nemit> : <ncontext> : <nbasepairs> : <basepair_type> : <e-name>
```

If for an emission distribution, we want to specify the different distributions, we add a number at the end. Example, an stacked basepair:

If no parameters values are going to be specified:

```
edist : 2 : 2 : 1 : _WW_ : e1
```

If parameter values are going to be added:

```
edist : 2 : 2 : 1 : _WW_ : e1 : :  0 # stacked on AA
NN = -p-INF
edist : 2 : 2 : 1 : _WW_ : e1 : :  1 # stacked on AC
NN = -p-INF
edist : 2 : 2 : 1 : _WW_ : e1 : :  2 # stacked on AG
NN = -p-INF
edist : 2 : 2 : 1 : _WW_ : e1 : :  3 # stacked on AU
NN = -p-INF
AU = 3
UA = 3
CG = 5
GC = 5
UG = 2 GU = 2
edist : 2 : 2 : 1 : _WW_ : e1 : :  4 # stacked on CA
NN = -p-INF
edist : 2 : 2 : 1 : _WW_ : e1 : :  5 # stacked on CC
NN = -p-INF
edist : 2 : 2 : 1 : _WW_ : e1 : :  6 # stacked on CG
NN = -p-INF
edist : 2 : 2 : 1 : _WW_ : e1 : :  7 # stacked on CU
NN = -p-INF
edist : 2 : 2 : 1 : _WW_ : e1 : :  8 # stacked on GA
NN = -p-INF
edist : 2 : 2 : 1 : _WW_ : e1 : :  9 # stacked on GC
NN = -p-INF
edist : 2 : 2 : 1 : _WW_ : e1 : :  10 # stacked on GG
NN = -p-INF
edist : 2 : 2 : 1 : _WW_ : e1 : :  11 # stacked on GU
NN = -p-INF
edist : 2 : 2 : 1 : _WW_ : e1 : :  12 # stacked on UA
NN = -p-INF
edist : 2 : 2 : 1 : _WW_ : e1 : :  13 # stacked on UC
NN = -p-INF
edist : 2 : 2 : 1 : _WW_ : e1 : :  14 # stacked on UG
NN = -p-INF
edist : 2 : 2 : 1 : _WW_ : e1 : :  15 # stacked on UU
NN = -p-INF
```

The “NN” value is the default (use “N” for a single emission, “NNN” for a triplet emission,...). After the default value (obligatory field if one adds values), one can specify other specific values, as in the example given above for the basepair distribution stacked on pair AU.

For a basepair emission that only allows A-U/C-G/G-U basepair combinations:

```
edist : <nemit> : <ncontext> : <nbasepairs> : <basepair_type> : wccomp : <e-name>
```

If the emission distribution is "silent" because the context is forbidden, *i.e.* a basepair stacked on a A-A pair:

```
edist : 2 : 2 : 1 : _WW_ : wccomp : e1 : : 1 : silent # stacked on AA
```

**The length distributions**

*Length distributions* need to specify a minimum length, a maximum length, and optionally a "fit" length at which point one assumes an extrapolated tail, and a name for the distribution. Possible distribution tails allowed are: "affine" which is used in thermodynamic models, and "linear" which in log space corresponds to assuming a geometric distribution tail. Length distribution names are of the form "l⟨n⟩" where ⟨n⟩ is a natural number.

Two types of length distribution are allowed: "monosegment" used for for instance for hairpin loops and bulges, and "disegment" (ldist-di) used for internal loops or stems. Each length distribution is associated with a single residue emission distribution that gets trained but cannot be tied to external emission distributions. For full disegment length distributions one also needs to specify the minimum number of residues for the left and right segments.

General description of a monosegment length distribution definition is:

```
ldist : <min> : <fit> : <max> : <l-name>
```

where "min" is the minimum length of the segment, "fit" is the length at which the distribution is fitted to a tail, and "max" is the maximum length of the distribution.
General description of a disegment length distribution definition is:

```
ldist-di : <minL> : <minR> sep <min> : <fit> : <max> : <l-name>
```

where "minL" is the minimum length of the left segment, "minR" is the minimum length of the right segment, and "min" is the minimum length of the sum of both segments.

An example in which some parameters values have been specified as in the thermodynamic model implemented by ViennaRNA 1.8.4 is:

```
ldist : 3 : p-D_FIT_HAIRPIN_LENGTH-2 : p-D_MAX_HAIRPIN_LENGTH-2 : l1 # hairpinloop's ldist
ld = -p-INF
3 = p-TT * p-hairpin37_length_5
4 = p-TT * p-hairpin37_length_6
5 = p-TT * p-hairpin37_length_7
6 = p-TT * p-hairpin37_length_8
7 = p-TT * p-hairpin37_length_9
8 = p-TT * p-hairpin37_length_10
9 = p-TT * p-hairpin37_length_11
10 = p-TT * p-hairpin37_length_12
```

```
# fit :  affine :  a :  b :  c :  d #corresponds to sc(x)=a+b*log(x*c+d)
# fit :  linear :  a :  b #corresponds to sc(x)=a+bx
fit :  affine :  p-TT * p-hairpin37_length_30 :  p-lxc :  1.0/p-D_FIT_HAIRPIN_LENGTH : 2.0/p-D_FIT_HAIRPIN_LENGTH
```

In this monsegment distribution (named "l1"), after the default value "ld =", 10 specific values have been specified. For lengths p-D_FIT_HAIRPIN_LENGTH-2 or larger we use an "affine" fit $sc(x) = a + b * log(x * c + d)$. A linear fit $sc(x) = a + xb$, which corresponds to a geometric fit for a probabilistic model, is also possible.
An example of a disegment length distribution with some assymetry parameters specified is:

```
ldist-di :  1 :  1 :  2 :  p-D_FIT_INTERNAL_LENGTH : p-D_MAX_INTERNAL_LENGTH : l3
ld,ld = p-ZERO
lsum = 1 = p-TT * p-internal_loop37_length_5
ldif = 1 += MAX(p-MAX_NINIO, p-TT * 1 * p-F_ninio37_2)
```

Here specific values have been assigned for particular values of the sum of the two segments ("lsum=1"), and the difference ("ldif = 1"). Values can be added (+=) or substracted (-=) as well.

**The rewrite rules** *Production rules* start with a single nonterminal to the left (as required formally by SCFGs), followed by an arrow "->" followed by an arbitrary number of terminals and nonterminals grouped into rules. A rule is a group of terminals and nonterminals executed together. The different rules associated to a nonterminal can be given all together connected by |'s (the "or" symbol), or in separate lines, or a combination of the two. The rules for a given nonterminal do not need to be consecutive, and they can appear in between the rules for other nonterminals.

Rules are composed of nonterminals and terminals. Nonterminals are represented by capital letters or capital letters followed by a natural number. Examples of valid nonterminals are:

```
S, S2, S21, P234^{p}, K^{pm}, H1^{abc},...
```

There are four types of terminals: residue terminals which produce a finite number of residues according to an emission distribution, monosegment and disegment terminals, which produce a variable number of residues according to a length distribution, and the "empty string" terminal. Residue terminals are represented by any lower-case letter with the exception of "`e`" which is reserved for the "empty string" terminal, and "`i`", "`j`", "`k`", and "`l`" which are reserved for iterators. Each monosegment terminal "`m...m(i,j)`" uses a monosegment length distribution. Disegment terminals "`d...(i,k) d...(l,j)`" can specify a disegment length distribution or a monosegment length distribution in which case TORNADO assumes that the argument of the distribution is the sum of the two segments. The special stem disegment terminal "`d...(i,k) d'...(l,j)`" is reserved to the emission of whole stems for which `k-i=j-l`. Stem disegments can be tied to external basepair or stacked basepair emission distributions.

## Detailed description of a particular grammar: ViennaRNAG

Here we describe the TORNADO code for a grammar that implements the standard nearest-neighbor model of nucleic acids interactions. This grammar when parameters are given some specific values reproduces the implementation of the standard package ViennaRNA 1.8.4. For simplicity, here we provide the grammar without any specific values. A version of the same grammar that includes the parameter values to reproduce results of ViennaRNA 1.8.4 is given in supplemental file “ViennaRNAG.grm”.

Comments outside the actual TORNADO code are given in red.

*comments use “#” or “//”*

```
# ViennaRNAGz
#
# ViennaRNAG without the scores.
```

*–FIRST come the parameters (not many in this case since here we don’t provide any specific parameter values).*

```
# ====================
# param definitions
# ====================

def : p-INF : 1000000
def : p-ZERO : 0.0

def : p-MAXLOOP                    : 30                          # p-MAXLOOP=30
def : p-D_FIT_HAIRPIN_LENGTH       : p-MAXLOOP                   # fit loop size for hairpin loops is 30
def : p-D_FIT_BULGE_LENGTH         : p-MAXLOOP                   # fit loop size for bulge loops is 30
def : p-D_FIT_INTERNAL_LENGTH      : p-MAXLOOP                   # fit loop size for internal loops loops is 30
def : p-D_MAX_HAIRPIN_LENGTH       : 4000                        # max loop size for hairpin loop is 400
def : p-D_MAX_BULGE_LENGTH         : p-D_FIT_BULGE_LENGTH        # max loop size for bulge loops is 3
def : p-D_MAX_INTERNAL_LENGTH      : p-D_FIT_INTERNAL_LENGTH     # max loop size for internal loops loops is 30
```

*–SECOND come the transition distribution definitions*

```
# ===========================
# transition distributions
# ===========================

tdist : 2 : t-F0        # distribution used by all F0^{±+} nonterminals
tdist : 2 : t-M2        # distribution used by all M2 nonterminals
tdist : 2 : t-M1        # distribution used by M1^{±+} nonterminals
tdist : 3 : t-M         # distribution used by M^{±±} nonterminals
tdist : 2 : t-L1        # distribution used by L1^{+±} nonterminals
tdist : 24 : t-P        # distribution used nonterminal P
tie : 3 : 4             # nonterminal P transitions to left and right bulges of same length are tied
tie : 5 : 6
tie : 7 : 8
tie : 10 : 11           # nonterminal P transitions to left and right internal loops of same total length are tied
tie : 14 : 15
tie : 17 : 18
```

*–THIRD come the emission distribution definitions*

```
# ==========================
# emission distributions
# ===========================
# ----------------------------
# unpaired [e1]
#
# P(i)
# ----------------------------
edist : 1 : 0 : 0 : e1      (single-base emission named e1)

# ----------------------------
# closing basepair [e1]
#
# P(i&j)
# ----------------------------
edist : 2 : 0 : 1 : _WW_ : e1      (Watson-crick cis basepair emission named e1)

# ----------------------------
# basepair [e2]
#
# P(i&j)
# ----------------------------
edist : 2 : 0 : 1 : _WW_ : e2      (Watson-crick cis basepair emission named e2)

# ------------------------------------------------------------------------------------------
# stacked base_pair [e1]
#
# P(i&j | i-1&j+1) = TT * p-stack37_(i-1)(j+1)(i)(j) + (1 - TT) * p-enthalpies_(i-1)(j+1)(i)(j)
#     (the comments above refer to the correspondence with parameters as defined in ViennaRNA 1.8.4 code)
# ------------------------------------------------------------------------------------------
edist : 2 : 2 : 1 : _WW_ : e1      (stacked basepair emission named e1)

# ----------------------------------------
# stacked closing basepair [e5]
#
# P(i&j | i-1&j+1)
# ----------------------------------------
edist : 2 : 2 : 1 : _WW_ : e5

# ---------------------------------------------------------------------------------------
# terminal_mismatch [e1]
# used in hairpin loops
```

```
#
#  P(i,j | i-1&j+1) = TT * p-mismatchH37_(i-1)(j+1)(i)(j) + (1 - TT) * p-mism_H_(i-1)(j+1)(i)(j)
#                 -p-TerminalAU (when it applies)
# ---------------------------------------------------------------------------------------------
edist : 2 : 2 : 0 : e1      (emission of two single bases dependent on closing bases)

# ---------------------------------------------------------------------------------------------
#  terminal_mismatch [e2]
#  used in internal loops
#
#  P(i,j | i-1&j+1) = TT * p-mismatchI37_(i-1)(j+1)(i)(j) + (1 - TT) * p-mism_H_(i-1)(j+1)(i)(j)
# ---------------------------------------------------------------------------------------------
edist : 2 : 2 : 0 : e2

# ------------------------------------------------
#  3-dangle [e1]
#
#  P(i | i-1&j+1) = p-dangle3_smooth_(j+1)(i-1)(i)
# ------------------------------------------------
edist : 1 : 2 : 0 : e1

# ------------------------------------------------
#  5-dangle [e2]
#
#  P(j | i-1&j+1) = p-dangle5_smooth_(j+1)(i-1)(j)
# ------------------------------------------------
edist : 1 : 2 : 0 : e2

# ---------------------------------------------------------------------------------------------------
#  dangle in 1nt bulge [e5]
#
#  P(j | i-1&j+1) = -p-TerminalAU, if not CG or GC # yes negative, we are removing a previously added term
#                         p-ZERO if CG or GC
# ---------------------------------------------------------------------------------------------------
edist : 1 : 2 : 0 : e5

          the distribution below is tied as a joint distribution. It assumes independence of the two dangles
# ---------------------------------------------------------------------------------------------------
#  multi_mismatch [e3]
#
#  P(i,j | i-1&j+1) = p-dangle3_smooth_(i-1)(j+1)(i) + p-dangle5_smooth_(i-1)(j+1)(j)
#
#  tied by JOINT: P(i,j | i-1&j+1) = P(i | i-1&j+1) * P(j | i-1&j+1)
#                                         e1_1_2         e2_1_2      (already defined distributions)
# ---------------------------------------------------------------------------------------------------
edist : 2 : 2 : 0 : e3
tied : e1_1_2 : 0 : e2_1_2 : 0 : joint

# ----------------------------------------------
#  tetraloops [e1]
#
#  < _ _ _ _ >
#
#  P(i, i+1, i+2, i+3 | i-1, i+4)
# ----------------------------------------------
edist : 4 : 2 : 0 : e1

          the distribution below is tied as a joint distribution. It assumes independence of the two dangles
# ----------------------------------------------
#  two dangles [e1]
#
#  P(i,j)
#
#  tied by JOINT: P(i,j) = P(i) * P(j)
#                          e1_1_0  e1_1_0      (already defined distributions)
# ----------------------------------------------
edist : 2 : 0 : 0 : e1
tied : e1_1_0 : 0 : e1_1_0 : 0 : joint

          the distribution below is tied by "rotation"
# ------------------------------------------------------------------------------
#  intloop_internal closing basepair dependent on L-R dangle [e2]
#
#  P(i&j | i-1,j+1)
#
#  tied by ROTATION: P(i&j | i-1,j+1) = P(j+1,i-1 | j&i) * P(j&i) / P(i-1,j+1)
#                                        e2_2_2         e1_2_0     e1_2_0      (already defined distributions)
# ------------------------------------------------------------------------------
edist : 2 : 2 : 1 : _WW_ : e2
tied : e2_2_2 : 0 : e1_2_0 : 1 : e1_2_0 : 0 : rotate

# ------------------------------------------------------------------------------
#  multiloop or external closing basepair dependent on L-R dangle [e3]
#
#  P(i&j | i-1,j+1)
#
#  tied by ROTATION: P(i&j | i-1,j+1) = P(j+1,i-1 | j&i) * P(j&i) / P(i-1,j+1)
#                                        e3_2_2         e1_2_0     e1_2_0      (already defined distributions)
```

```
# ------------------------------------------------------------------------------------------
edist : 2 : 2 : 1 : _WW_ : e3
tied : e3_2_2 : 0 : e1_2_0 : 1 : e1_2_0 : 0 : rotate

# ------------------------------------------------------------------------------------------
#  1x1 internal loops with closing pair, dependent on previous pair[e1]
#
#    <   -   < [ ]   >   -   >
#     .   .    . [ ]   .    .    .
#    i-1  i    i+1    j-1    j   j+1
#     f    a     e      e'    g    f'
#
#  P(a^g | f^f' e^e')
#
# ------------------------------------------------------------------------------------------
edist : 2 : 4 : 0 : e1

# ------------------------------------------------------------------------------------------
#  1x2 internal loops with closing pair, dependent on previous pair[e1]
#
#    <   -   < [ ]   >   -   -   >
#     .   .    . [ ]   .    .    .    .
#    i-1  i    i+1    j-2    j-1   j   j+1
#     f    a     e      e'    c     g    f'
#
#  P(a^cg | f^f' e^e')
#
# ------------------------------------------------------------------------------------------
edist : 3 : 4 : 0 : e1

# ------------------------------------------------------------------------------------------
#  2x2 internal loops with closing pair, dependent on previous pair [e1]
#
#    <   -   -   < [ ]   >   -   -   >
#     .   .   .    . [ ]   .    .    .    .
#    i-1  i  i+1   i+2    j-2    j-1   j   j+1
#     f    a    b    e      e'    c     g    f'
#
#  P(ab^cg  | f^f' e^e')
#
# ------------------------------------------------------------------------------------------
edist : 4 : 4 : 0 : e1
```

*–FORTH come the loop length distribution definitions*

```
# ==========================================================================================
#  length distributions
# ==========================================================================================
```

*three monosegment distribution*

```
ldist : 3 : p-D_FIT_HAIRPIN_LENGTH-2 : p-D_MAX_HAIRPIN_LENGTH-2 : l1        # hairpin loop length distribution
ldist : 2 : p-D_FIT_BULGE_LENGTH : p-D_MAX_BULGE_LENGTH : l2                # bulges length distribution
ldist : 2 : p-D_FIT_INTERNAL_LENGTH-2 : p-D_MAX_INTERNAL_LENGTH-2 : l7  # internal loops length distribution for the particular case: 1x(>2) and (>2)x1
```

*one disegment distribution for generic internal loops*

```
ldist-di : 0 : 0 : 1 : p-D_FIT_INTERNAL_LENGTH-4 : p-D_MAX_INTERNAL_LENGTH-4 : l3 # internal loops length distribution
```

*–LAST come the rules*

```
# ==========================================================================================
#  The basic ViennaRNA grammar rules are:
#
#  S --> S a  | S F0  | e
#  F0 --> a:i&j e1 F5(i+1,j-1)  | a:i&j e1 P(i+1,j-1)
#  F5 --> a:i&j:i-1,j+1 e1 F5(i+1,j-1)  | a:i&j:i-1,j+1 e1 P(i+1,j-1)
#  P --> m...m l1  | m...m F0 l2  | F0 m...m l2  | d... F0 ...d l3  | M2
#  M2 --> M M1
#  M --> M M1  | L1
#  M1 --> M1 a e1  | F0
#  L1 --> a e1 L1  | M1
#
#  Equivalences with the names given in ViennaRNA 1.8.4 code (part_func.c):
#
#  S <--> q
#  F0 <--> qq
#  F5 <--> qb
#  M <--> qm
#  M1 <--> qqm
#
# ==========================================================================================

# ==================
#  rules
# ==================
```

$$S \longrightarrow S^{+} a \quad | \quad S^{-} \mid \quad e$$

```
S –> S∧{p}(i,j-1) a:j e1  | S∧{m}  | e                                         # this first rule defines "S" as the start nonterminal

           S^+    ⟶ S^+ a   |  S^+  a   F0^{++}  |  S^-  F0^{-+}  |  F0^{-+} |  e

S∧{p} –> t-S∧{p}   S∧{p}(i,j-1) a:j e1
S∧{p} –> t-S∧{p}   S∧{p}(i,k-1) a:k e1 F0∧{pp}(k+1,j)
S∧{p} –> t-S∧{p}   S∧{m}(i,k) F0∧{mp}(k+1,j)
S∧{p} –> t-S∧{p}   F0∧{mp}(i, j)
S∧{p} –> t-S∧{p}   e

           S^-    ⟶ S^+  a   F0^{+-}  |  S^- F0^{--}  |  F0^{--}
S∧{m} –> t-S∧{m}   S∧{p}(i,k-1) a:k e1 F0∧{pm}(k+1,j)
S∧{m} –> t-S∧{m}   S∧{m}(i,k) F0∧{mm}(k+1,j)
S∧{m} –> t-S∧{m}   F0∧{mm}(i, j)

# ————————————————————————————————————————————
# HELIX
#
# F0 starts a external helix.
#
#  A external basepair can depend on dangles:
#  none F0∧{mm}
#  one F0∧{pm} and F0∧{mp}
#  two F0∧{pp}
# ————————————————————————————————————————————

          F0^{αβ}   ⟶ a   F5  a'   |  a   P  a'
F0∧{pp}    –> t-F0   a:i&j:i-1,j+1  e3 F5(i+1,j-1)  | a:i&j:i-1,j+1  e3 P(i+1,j-1)       # basepair + L-dangle + R-dangle
F0∧{pm}    –> t-F0   a:i&j:i-1       e1 F5(i+1,j-1)  | a:i&j:i-1       e1 P(i+1,j-1)       # basepair + L-dangle
F0∧{mp}    –> t-F0   a:i&j:j+1       e2 F5(i+1,j-1)  | a:i&j:j+1       e2 P(i+1,j-1)       # basepair + R-dangle
F0∧{mm}    –> t-F0   a:i&j           e1 F5(i+1,j-1)  | a:i&j           e1 P(i+1,j-1)       # basepair

# ————————————————————————————————————————————
# F5 continues a helix adding the stacking for each new pair
#
# ————————————————————————————————————————————

           F5     ⟶ a   F5  a'   |  a   P  a'
F5 –> a:i&j:i-1,j+1 e1 F5(i+1,j-1)  | a:i&j:i-1,j+1 e5 P(i+1,j-1)

# ————————————————————————————————————————————
# G0∧{pp} is like FO∧{pp} but for starting helices inside a internal loop
#
#  the difference is that it uses mismatchI37 instead of dangles
# ————————————————————————————————————————————

          G0^{++}   ⟶ a   F5  a'   |  a   P  a'
G0∧{pp} –> a:i&j:i-1,j+1 e2 F5(i+1,j-1)  | a:i&j:i-1,j+1 e2 P(i+1,j-1)

# ——————————————————————————
# P–>HAIRPINLOOP
#  0,1,2 nt hairpin loops forbidden
#
#  abc
#  abcd
#  a m..m b
# ——————————————————————————

            P     ⟶ a   b   c   |  a   b   c   d   |  a   m...m b
P –> t-P   a:i e1 b:i+1 e1 c:i+2 e1                     # Triloops
P –> t-P   a:i,i+1,i+2,i+3:i-1,j+1 e1                   # Tetraloops
P –> t-P   a:i,j :i-1,j+1 e1 m...m(i+1,j-1) l1           # hairpin loops >= 5nts

# ————————————————————————————————————
# P–>BUlGES
#  (no dangles at all)
#
#  b a {F5 | P} a'
#  a {F5 | P} a' c #a∧a' stacked on previous bp
#  m...m F0
#  F0 m..m
# ————————————————————————————————————

            P     ⟶ b   a   F5 a'  |  a  F5 a' c
P –> t-P   b:i:i-1,j+1 e5 a:i+1&j:i-1,j+1 e1  F5(i+2,j-1)                               # 1x0 bulges
P –> t-P                                       F5(i+1,j-2)  a:i&j-1:i-1,j+1 e1 c:j:i-1,j+1 e5   # 0x1 bulges

            P     ⟶ b   a   P a'  | a   P a'  c
P –> t-P   b:i:i-1,j+1 e5 a:i+1&j:i-1,j+1 e5  P (i+2,j-1)                               # 1x0 bulges
P –> t-P                                       P (i+1,j-2)  a:i&j-1:i-1,j+1 e5 c:j:i-1,j+1 e5   # 0x1 bulges

            P     ⟶ m...m F0^{--}  |  F0^{--}   m...m
P –> t-P   m...m(i,k) l2        F0∧{mm}(k+1,j)
P –> t-P                        F0∧{mm}(i,l-1)    m...m(l,j) l2

# ————————————————————————————————————
# P–>INTERNAL LOOPS
#
```

```
#  a e {F5 | P} e' g # 1x1
#  a e {F5 | P} e' c g # 1x2
#  a b e {F5 | P} e' g # 2x1
#  a b e {F5 | P} e' c g # 2x2
#
#  a G0 m...m g # (l1= 1)x(l2> 2)
#  a m...m G0 g # (l1> 2)x(l2= 1)
#  a d... b G0 c ...d g #(l1>=2)x(l2>=2) and l1+l2 > 4
#
# ------------------------------------------------------------------------------

          P    ⟶ a  e  F5 e' g  | a  e  F5 e' c  g  | a  b  e  F5 e' g  | a  b  e  F5 e' c  g
P –> t-P   a:i+1&j-1 e2 b:i, j:i-1,j+1,i+1,j-1 e1          F5(i+2,j-2)       # 1x1
P –> t-P   a:i+1&j-2 e2 b:i, j-1,j:i-1,j+1,i+1,j-2 e1      F5(i+2,j-3)       # 1x2
P –> t-P   a:i+2&j-1 e2 b:j, i,i+1:i-1,j+1,i+2,j-1 e1      F5(i+3,j-2)       # 2x1 (need to reverse the order here)
P –> t-P   a:i+2&j-2 e2 b:i,i+1,j-1,j:i-1,j+1,i+2,j-2 e1   F5(i+3,j-3)       # 2x2          #

          P    ⟶ a  e  P e' g  | a  e  P e' c  g  | a  b  e  P e' g  | a  b  e  P e' c  g
P –> t-P   a:i+1&j-1 e1 b:i, j:i-1,j+1,i+1,j-1 e1          P (i+2,j-2)       # 1x1
P –> t-P   a:i+1&j-2 e1 b:i, j-1,j:i-1,j+1,i+1,j-2 e1      P (i+2,j-3)       # 1x2
P –> t-P   a:i+2&j-1 e1 b:j, i,i+1:i-1,j+1,i+2,j-1 e1      P (i+3,j-2)       # 2x1 (need to reverse the order here)
P –> t-P   a:i+2&j-2 e1 b:i,i+1,j-1,j:i-1,j+1,i+2,j-2 e1   P (i+3,j-3)       # 2x2

          P    ⟶ a  m...m G0^{++} g  | a  G0^{++} m...m g
P –> t-P   a:i,j:i-1,j+1 e2 m...m(l,j-1) l7                G0∧{pp}(i+1,l-1)  # (l1= 1)x(l2> 2)
P –> t-P   a:i,j:i-1,j+1 e2 m...m(i+1,k) l7                G0∧{pp}(k+1,j-1)  # (l1> 2)x(l2= 1)

          P    ⟶ a  d... b  G0^{++} c  ...d g
P –> t-P   a:i,j:i-1,j+1 e2 b:k,l e1 d...(i+1,k-1)...d(l+1,j-1) l3 G0∧{pp}(k+1,l-1)    #  (l1>=2)x(l2>=2) and l1+l2 > 4

# ------------------------------------------------------------------------------------------
#  P–>MULTILOOPS
#
#  In principle one only needs 3 NTs here:
#
#  M2 = multiloop with at least 2 helices
#  M = multiloop with at least 1 helix
#  M1 = a helix with possibly some unpaired bases to the right of the helix
#
#  the basic (unambiguous) recursion is:
#  P –> M2
#
#  M2 –> M M1
#  M1 –> M1 a  | F0
#  M –> M M1  | L1
#  L1 –> a L1  | M1
#
#  but because the energy model likes to add contributions for dangles
#  we need to keep track of when Mx has already generated that dangle or not.
#  The convention is:
#
#  Mx∧{pp} == L/R-dangles have been generated. It can freely add more bases in both sides
#  Mx∧{mp} == R-dangle has been generated. It can freely add more bases R but not L
#  Mx∧{pm} == L-dangle has been generated. It can freely add more bases L but not R
#  Mx∧{mm} == No dangles have been generated. No free bases can be added L or R.
#
# ------------------------------------------------------------------------------------------

          P    ⟶  a  M2^{++}  b  | a   M2^{+-}   |  M2^{-+}  b  | M2^{--}
P –> t-P   a:i,j:i-1,j+1 e3 M2∧{pp}(i+1,j-1)  | a:i:i-1,j+1 e1 M2∧{pm}(i+1,j)  | M2∧{mp}(i,j-1) a:j:i-1,j+1 e2  | M2∧{mm}

        M2^{αβ}   ⟶  M^{α+} a    M1^{+β} | M^{α-} M1^{-β}
M2∧{pp}    –> t-M2     M∧{pp}(i,k-1) a:k e1 M1∧{pp}(k+1,j)    |  M∧{pm} M1∧{mp}
M2∧{pm}    –> t-M2     M∧{pp}(i,k-1) a:k e1 M1∧{pm}(k+1,j)    |  M∧{pm} M1∧{mm}
M2∧{mp}    –> t-M2     M∧{mp}(i,k-1) a:k e1 M1∧{pp}(k+1,j)    |  M∧{mm} M1∧{mp}
M2∧{mm}    –> t-M2     M∧{mp}(i,k-1) a:k e1 M1∧{pm}(k+1,j)    |  M∧{mm} M1∧{mm}

        M1^{α+}   ⟶  M1^{α+} a    | F0^{α+}
M1∧{pp}    –> t-M1     M1∧{pp}(i,j-1) a:j e1   |  F0∧{pp}
M1∧{mp}    –> t-M1     M1∧{mp}(i,j-1) a:j e1   |  F0∧{mp}

        M1^{α-}   ⟶  F0^{α-}
M1∧{pm}    –>          F0∧{pm}
M1∧{mm}    –>          F0∧{mm}

        M^{αβ}    ⟶ M^{α+}  a    M1^{+β} | M^{α-} M1^{-β} | L^{αβ}
M∧{pp}     –> t-M      M∧{pp}(i,k-1) a:k e1 M1∧{pp}(k+1,j)   | M∧{pm} M1∧{mp}   |  L1∧{pp}
M∧{pm}     –> t-M      M∧{pp}(i,k-1) a:k e1 M1∧{pm}(k+1,j)   | M∧{pm} M1∧{mm}   |  L1∧{pm}
M∧{mp}     –> t-M      M∧{mp}(i,k-1) a:k e1 M1∧{pp}(k+1,j)   | M∧{mm} M1∧{mp}   |  M1∧{mp}
M∧{mm}     –> t-M      M∧{mp}(i,k-1) a:k e1 M1∧{pm}(k+1,j)   | M∧{mm} M1∧{mm}   |  M1∧{mm}

        L^{+β}    ⟶ a     L^{+β}    | M1^{+β}
L1∧{pp}    –> t-L1     a:i e1 L1∧{pp}(i+1,j)   |  M1∧{pp}
L1∧{pm}    –> t-L1     a:i e1 L1∧{pm}(i+1,j)   |  M1∧{pm}
```

# 4 Inference programs implemented in TORNADO

## Obtaining properties of and RNA grammar and debugging tool: `grm-parse`

Program `grm-parse` produces an extensive description of the properties of the grammar: number of transition, emission and length distributions, enumeration of rules, which distributions are used by a given rule, and more.

Example is:

```
bin/grm-parse grammars/ViennaRNAG.grm
```

`grm-parse` has three major other uses in addition to getting information about a grammar:

- It is the debugging tool when constructing a grammar in TORNADO language. `grm-parse` would stop if the grammar description does not follow the TORNADO language specifications. Using option `-v`, one can see where the parsing of the grammar failed.
- It can be used to consolidate the counts of different maximum likelihood training sets into one. Example:

```
bin/grm-parse --count --countsavefile examples/TrainSetATrainSetB_ViennaRNAG.counts //
        grammars/ViennaRNAG.grm examples/TrainSetA_ViennaRNAG.counts //
        examples/TrainSetB_ViennaRNAG.counts

bin/grm-parse --count --countsavefile examples/TrainSetATrainSetBTrainSetB_ViennaRNAG.counts //
        grammars/ViennaRNAG.grm examples/TrainSetA_ViennaRNAG.counts //
        examples/TrainSetB_ViennaRNAG.counts examples/TrainSetB_ViennaRNAG.counts
```

- It can be used to extract the set of parameter values for a grammar, when those have been given as part of the grammar description file. Example:

```
bin/grm-parse --scoresavefile examples/ViennaRNAG_thermo.scores grammars/ViennaRNAG.grm
```

Complete list of options:

- `-v`: be verbose.
- `--bck`: report backward rules (used with the outside algorithm).
- `--count`: grammar paramfile is given as counts.
- `--lprob`: grammar paramfile is given as logprobs.
- `--score`: grammar paramfile is given as scores.
- `--distcounts`: report counts per distribution.

- --cweightfile <s>: for multiple training sets: read training set weights from <s>.
- --countsavefile <s>: save score file to <s>.
- --paramsavefile <s>: save param file to <s>.
- --scoresavefile <s>: save score file to <s>.
- --margsavefile <s>: save marginals for the distributions of the grammar to <s>.

## Training: grm-train

Training of a grammar is performed by program grm-train and uses the maximum likelihood method (ML). It requires a file describing the grammar and a Stockholm-formatted file with the trusted individual sequences and their structures.

A typical command line is (from TORNADO's main directory):

```
bin/grm-train grammars/ViennaRNAG.grm data/CG/sto/S-151Rfam.sto //
        examples/S-151Rfam_ViennaRNAG.param
```

This command line produces file "S-151Rfam_ViennaRNAG.param" with probabilistic parameters for the grammar "grammars/ViennaRNAG.grm", based on the structures of "data/CG/sto/S-151Rfam.sto". (Other real examples can be found in directory examples/.)

Important options are:

- --countsavefile <file>: allows to store the parameters in count form, which is very convenient in order to combine different training sets into one.

  ```
  bin/grm-train --countsavefile examples/S-151Rfam_ViennaRNAG.counts grammars/ViennaRNAG.grm
  //
          data/CG/sto/S-151Rfam.sto example/S-151Rfam_ViennaRNAG.param
  ```

- --mpi: uses a Message Passing Interface implementation for use in clusters.
- --margsavefile <file>: saves to <file> the marginal (A/C/G/U) probabilities of all the emission distributions of the grammar.
- --null <file>: saves to <file> a first-order Markov base-composition (A/C/G/U) model for the training set.

## Testing: grm-fold

RNA secondary structure prediction of an RNA sequence given a grammar and a set of parameters values is performed by program grm-fold. It requires a file describing the grammar and a file (in fasta or Stockholm format) with the RNA sequences to fold.

A typical command line is (from TORNADO's main directory):

```
bin/grm-fold --score grammars/ViennaRNAG.grm examples/test.sto //
        examples/test_ViennaRNAG_prob.sto
```

It produces a Stockholm formatted file ("TestSetA_ViennaRNAG.sto") with the secondary structure predictions for test set "data/TORNADO_RNA2011_benchmark/TORNADO_TestA.sto", given the grammar "grammars/ViennaRNAG.grm" that includes thermodynamic scores (emulation of ViennaRNA 1.8.4) inside the file. By default, it uses the CMEA method by Do *et al.*, 2006.

If you want to override the parameter values in the grammar file and use other set of values (for instance counts contained in file "S-151Rfam_ViennaRNAG.counts", also if the grammar file does not include any specific values)

```
bin/grm-fold --count grammars/ViennaRNAG.grm examples/test.sto //
        examples/test_ViennaRNAG_prob.sto //
        examples/TrainSetA_ViennaRNAG.counts
```

If parameter values are given as "count" (and only in that case), more than one set of counts can be added in the command line. The probabilities of the parameters will be calculated after summing the counts provided by all count sets.

Example:

```
bin/grm-fold --count grammars/ViennaRNAG.grm examples/test.sto //
        examples/test_ViennaRNAG_prob_AB.sto //
        examples/TrainSetA_ViennaRNAG.counts //
        examples/TrainSetB_ViennaRNAG.counts
```

Options to select the type of parameter values used by the grammar:

- `--count`: Default. Grammar parameter values are provided as scores.
- `--lprob`: Grammar parameter values are provided as probabilities.
- `--score`: Grammar parameter values are provided as counts.

Alternative options to select the folding algorithm:

- `--cyk`:
- `--cmea`: (compatible with `--auc`)
- `--gcentroid`: (compatible with `--auc`)
- `--centroid`:

Other folding options

- `--auc`: can be used in combination with `--cmea` or `--gcentroid` in order to produce a collection of predicted structures depending on one parameters which can be tuned with options `--auc_l2min,--auc_l2max`.

```
bin/grm-fold --auc grammars/ViennaRNAG.grm examples/test.sto //
        examples/test_ViennaRNAG_prob_auc.sto examples/TrainSetA_ViennaRNAG.counts
```

- `--gpostfile`: changes the underlying grammar used to calculate the MEA structure. Default is "grammars/gmea_g6/gmea_g6.grm", which corresponds to $\gamma = 1$.
- `--force_min_loop`: allows to change the minimum hairpin loop size allowed by the grammar.
- `--force_min_stem`: allows to change the minimum stem (or helix) size allowed by the grammar.

Other options

- `--mpi`: uses a Message Passing Interface implementation for use in clusters .
- `--tsqfile <file>`: saves to file the input sequences (and structures if any) in the same order that they have been evaluated. This is useful to compare trusted to predicted structures when running MPI since the order of reported structures does not have to be the same as in the input file, and when reporting multiple predictions for a sequence using `--auc`.

```
bin/grm-fold --auc --tsqfile examples/test_ViennaRNAG_tprob_auc.sto //
        grammars/ViennaRNAG.grm examples/test.sto //
        examples/test_ViennaRNAG_prob_auc.sto examples/TrainSetA_ViennaRNAG.counts
```

### Comparing a trusted with a predicted structure: `esl-compstruct`

The easel library (included with the TORNADO package) allows us to compare two different structures for a given sequence. The application `esl-compstruct` calculates sensitivity and positive predictive value per sequence and for the whole set of sequences.

Examples are:

```
bin/easel/miniapps/esl-compstruct examples/test.sto examples/test_ViennaRNAG_prob.sto

bin/easel/miniapps/esl-compstruct examples/test.sto examples/test_ViennaRNAG_prob.sto

bin/easel/miniapps/esl-compstruct examples/test_ViennaRNAG_tprob_auc.sto //
        examples/test_ViennaRNAG_prob_auc.sto
```

### Calculate the score (or log probability) of a sequence/structure pair: `grm-score`

Program `grm-score` allows for a given sequence and structure to calculate the score for a given grammar and a set of parameter values. Parameter values can be thermodynamic, probabilistic or arbitrary scores. The folding options are identical to `grm-fold`, with CMEA as default.

Examples are:

To calculate the score of the probabilistic predictions "examples/test_ViennaRNAG_prob.sto" using the thermodynamic parameters:

```
bin/grm-score --score grammars/ViennaRNAG.grm examples/test_ViennaRNAG_prob.sto
```

To calculate the score of the thermodynamic predictions "examples/test_ViennaRNAG_thermo.sto" using the probabilistic parameters of "examples/TrainSetA_ViennaRNAG.counts":

```
bin/grm-score --count grammars/ViennaRNAG.grm //
        examples/test_ViennaRNAG_thermo.sto //
        examples/TrainSetA_ViennaRNAG.counts
```

### Sampling suboptimal structures from a probabilistic grammar: `grm-psample`

Program `grm-psample` allows for a given sequence to sample suboptimal structures from the posterior distribution.

Example that will produce 10 samples per sequence in file "examples/test.sto" is:

```
bin/grm-psample -n 10 --count grammars/ViennaRNAG.grm examples/test.sto //
        examples/test_ViennaRNAG_psample.sto //
        examples/TrainSetA_ViennaRNAG.counts
```

### Emitting sequence/structure pairs from a probabilistic grammar: `grm-emit`

Program `grm-emit` allows to generate directly from the SCFG sequences and structures.

Here is an example that will produce 100 sequences with their corresponding structures according to grammar "grammars/ViennaRNAG.grm" parameterized with the counts 'examples/TrainSetA_ViennaRNAG.counts":

```
bin/grm-emit -n 100 --count grammars/ViennaRNAG.grm//
        examples/ViennaRNAG_emit.sto //
        examples/TrainSetA_ViennaRNAG.counts
```

## References

Leontis, N. B. and Westhof, E. (2001). Geometric nomenclature and classification of RNA base pairs. *RNA*, 7:499–512.
